# Challenges in Measuring AMH in the Clinical Setting

**DOI:** 10.3389/fendo.2021.691432

**Published:** 2021-05-24

**Authors:** Hang Wun Raymond Li, David Mark Robertson, Chris Burns, William Leigh Ledger

**Affiliations:** ^1^ Department of Obstetrics and Gynaecology, The University of Hong Kong, Queen Mary Hospital, Pokfulam, Hong Kong; ^2^ Discipline of Obstetrics & Gynaecology, School of Women’s & Children’s Health, University of New South Wales, Sydney, NSW, Australia; ^3^ Biotherapeutics Division, National Institute for Biological Standards and Control, Potters Bar, United Kingdom

**Keywords:** anti-Müllerian hormone, enzyme-linked immunosorbent assay (ELISA), automated chemiluminescence immunoassay, reference preparation, international standard

## Abstract

Serum anti-Mullerian hormone (AMH) is a widely used marker of functional ovarian reserve in the assessment and treatment of infertility. It is used to determine dosing of gonadotropins used for superovulation prior to *in vitro* fertilization, as well as to determine the degree of damage to ovarian reserve by cytotoxic treatments such as chemotherapy. AMH is also now used to predict proximity to menopause and potentially provides a sensitive and specific test for polycystic ovarian syndrome. Twenty one different AMH immunoassay platforms/methods are now commercially available. Of those compared, the random-access platforms are the most reliable. However, to date there has not been an agreed common international AMH reference preparation to standardize calibration between the various immunoassays. Recently, a purified human AMH preparation (code 16/190) has been investigated by the World Health Organization as a potential international reference preparation. However, this was only partially successful as commutability between it and serum samples was observed only in some but not all immunoassay methods. Development of a second generation reference preparation with wider commutability is proposed.

## Introduction

Anti-Mullerian hormone (AMH), also known as Mullerian-inhibiting substance (MIS), was first recognized in the 1940s as the factor which determines regression of the Mullerian duct in the male fetus although it was only formally characterized and cloned in the 1980s (1, 2). Its production in the adult female was first reported in 1990 (3). With the development of serum AMH immunoassays it has become apparent that AMH is a clinically useful marker of functional ovarian reserve (4, 5) and thus of clinical value in the treatment of infertility, where measuring the follicle reserve is important.

Serum AMH is now used as a diagnostic test in infertile women undergoing controlled ovarian stimulation as part of an *in vitro* fertilization (IVF) program (6) and in the assessment of polycystic ovarian syndrome (PCOS), risk of ovarian hyperstimulation syndrome, prediction of menopause and monitoring the impact of cytotoxic chemotherapy and radiotherapy on ovarian function. Currently there are commercial kits available from more than 14 manufacturers (7, 8). However, the absence of an agreed international AMH reference preparation has resulted in confusion in defining clinical reference ranges between different kits. The aim of this report is to describe the development of AMH immunoassays and AMH reference preparations, and discuss the recently described WHO AMH Reference Reagent for immunoassay standardization.

## AMH Gene and Molecular Structure

AMH is a member of the transforming growth factor-beta (TGF- ß) superfamily. It is a 140 kDa homodimeric glycoprotein consisting of two identical glycoprotein subunits linked by disulphide bonds. In humans, AMH is encoded by the *AMH* gene, which is located on chromosome 19 p13.3. The *AMH* gene is 275 bp in length and consists of five exons. The GC-rich 3’ end of the fifth exon codes for the bioactive part of the AMH molecule (8, 9).

The *AMH* gene encodes a pre-protein of 560 amino acid residues (pre-proAMH) which is cleaved to produce the precursor (proAMH) (AMH_25-560_) that has no binding to the AMH receptor. It undergoes proteolytic cleavage by subtilisin/kexin proprotein convertases to the bioactive form, AMH_N,C_. AMH_N,C_ is a complex consisting of the N-terminal fragment (AMH_N_) and the C-terminal fragment (AMH_C_) associated non-covalently. The AMH_N_ fragment is a 110 kDa homo-dimer formed by two 57kDa subunits, whereas the AMH_C_ fragment is a 25 kDa homo-dimer formed by two 12.5 kDa subunits. Only AMH_N,C_ and AMH_C_ are bioactive on AMH receptors (**Supplementary Figure 1**). ProAMH and AMH_N,C_ are the circulating forms detectable in the blood in varying ratios, whereas the free AMH_C_ and AMH_N_ are not detectable in the blood circulation in physiological conditions. Current commercially available immunoassays detect both proAMH and AMH_N,C_, and the reported values are a composite of both. The physiological role of proAMH in the circulation is currently not clear (10–15).

## Correlation of Serum AMH with Ovarian Reserve

Although the primordial follicle count is conceptually the definitive parameter representing the ovarian reserve in a woman, it can only be measured directly by histological examination of the whole ovary after oophorectomy. Hence, surrogate markers which correlate with the primordial follicle count have been explored for clinical use, and both the AMH measured in serum and the antral follicle count (AFC) measured sonographically have been demonstrated to serve this purpose.

One study of 42 women undergoing oophorectomy for benign gynaecological conditions revealed a significant correlation (p<0.0001) between serum AMH and the ovarian primordial follicle count determined histologically, both unadjusted (r=0.72) as well as after adjustment for chronological age (r=0.48); the correlation coefficients were similar between AFC and primordial follicle count (unadjusted r=0.78 and adjusted r=0.53) (16). Most of the available studies showed a high correlation between serum AMH level and AFC (17–19).

## Intra- and Inter-Cycle Variations of Serum AMH

Most studies have demonstrated small fluctuations in serum AMH across the normal menstrual cycle with a decline in the late follicular phase. This pattern has been explained by a decrease in AMH secretion from the lead follicle as it achieves dominance prior to ovulation. However, the magnitude of these intra-cycle fluctuations is small and is not generally considered clinically relevant (20–24). One study revealed that the intra-cycle fluctuations remained within the same quintile in 72% of women and crossed two quintiles in only 1% of women (22). Two prospective studies (22, 25) explored the inter-cycle variability and suggested that between-cycle reproducibility of serum AMH is higher than that of serum FSH or AFC, and that only 11% of the variability resulted from intra-individual fluctuations between cycles (intra-class coefficient 0.89).

There are a small number of situations in which the intracycle fluctuation in AMH should be taken into account when assessing ovarian reserve. In particular, late reproductive aged women have a reduced number of follicular waves through the cycle, and hence AFC may be low and serum AMH can show marked changes (26) paralleling the pattern of the follicular waves. Similarly, following chemotherapy, the antral follicle reserve may be severely reduced and serum AMH profile may vary across the cycle or treatment period. In cases where the ovarian reserve is low, the more sensitive AMH immunoassays (sensitivity ~0.1ng/ml) are needed. Depending on its application, standardising the serum collection time in the cycle would appear to be a wise prerequisite in some situations. Sample collection alongside FSH and LH in the early follicular phase of the cycle allows for standardisation of timing in such situations. If the woman does not have a natural cycle then a random sample would be acceptable.

## Clinical Applications

A number of clinical situations have been identified in which serum AMH can be a useful diagnostic marker.

### a) Ovarian Reserve Testing

Prediction of ovarian response to superovulation is the most common application of serum AMH (6, 27). Two individual patient data meta-analyses (28, 29) have shown that both serum AMH and AFC had good performance in predicting poor ovarian response as well as excessive response. Ovarian stimulation regimes are now individualised to provide the optimum number of oocytes while avoiding risk of severe OHSS (30). In the IVF context, low oocyte yield in “poor responder” patients inevitably results in a smaller pool of cryopreserved embryos, thereby reducing the cumulative livebirth rate (LBR) from one IVF cycle, whereas larger numbers of eggs/embryos offers a higher cumulative LBR.

### b) PCOS

It is now well recognised that serum AMH is elevated in women with PCOS (5, 31, 32). In a recent study, AMH exhibited high specificity:sensitivity based on the receiver-operating characteristic (ROC) curve in predicting PCOS compared with age-matched controls (32).

### c) Prediction of Menopause

Several studies (33–35) have explored the use of single or multiple AMH samples over time as a means to predict menopause. Studies by Finkelstein (35) assessed the probability of AMH predicting menopause in women in the late reproductive age (~47y) and showed that in combination with age and body mass index, AMH measurement predicted the occurrence of menopause within 12 to 36 months (area under the ROC curve = 0.88-0.99). These conclusions were derived from an ultrasensitive AMH enzyme-linked immunosorbent assay (ELISA) with a lower detection limit of <2pg/ml. Assessment in women over a longer lead time (14 years) showed an improved prediction of menopause when including knowledge of the AMH decline rate (34).

### d) Monitoring the Return of Fertility in Those Women With Cancer Treated With Chemotherapy

Recovery of fertility in women following chemotherapy is a poorly defined area with evidence of shortened reproductive lifespan and infertility (3). Using AMH to monitor this process has revealed a complex pattern of recovery which is dependent both on the type of chemotherapy used and the woman’s age at treatment (36–38). In the study of Su et al. (38) dried blood analyses were undertaken using validated ELISA methodology with a sensitivity of 30pg/ml. The AMH level post treatment was 140pg/ml requiring sensitive ELISAs. Only 7% of samples were undetectable in this study.

### e) Serving as a Tumour Marker for Some Cancers

AMH can serve as a tumour marker for the detection or follow-up for recurrence of granulosa cell tumors (39–41).

## Evolution of AMH Assay Methods

Measurement of AMH in serum from adult women using ELISA was first reported in 1990 (3, 42). In the early 2000’s, when clinical studies utilizing AMH measurement were initiated, two commercial AMH ELISAs were available, manufactured by Diagnostic Systems Laboratories, Inc. (DSL, Webster, Texas, USA) and Immunotech (Marseille, France). DSL and Immunotech were subsequently acquired by Beckman Coulter, Inc. with the development of a second generation ELISA under the name “AMH Gen II ELISA”. This ELISA utilized the antibodies from the DSL kit and the AMH reference preparations from the Immunotech kit (43). Following its introduction in 2010, the AMH Gen II ELISA became the most widely used assay for AMH. However, its reliability was questioned due poor assay reproducibility, particularly following sample dilution and sample storage under different conditions (5, 44). The poor reproducibility was subsequently attributed to assay interference due to binding of serum complement protein C1q to the capture antibody. A pre-mixing protocol was then recommended by the manufacturer to overcome this problem. It was postulated that pre-mixing the test sample with the highly anionic buffer inactivated complement, hence reducing the interference. However, serum AMH values generated by the pre-mixing protocol are significantly higher compared with the conventional protocol (45).

More recently, additional AMH immunoassay kits have become available, including the ultrasensitive AMH/MIS ELISA kit (Ansh Laboratories, Texas, USA), the automated Access AMH kit (Beckman-Coulter Diagnostics, USA) and Elecsys^®^ AMH Immunoassay (Roche Diagnostics International Ltd, Indiana, USA). The latter two are automated immunoassays that utilize chemiluminescence for detection and are not susceptible to interference by serum complement (46). **Table 1** lists the analytical characteristics of the common commercial AMH assays that are currently available. Additional new AMH immunoassays are presented in the recent article by Ferguson et al. (8).

**Table 1:** Analytical characteristics of the common commercial AMH assay methods according to information from the manufacturers.

Characteristic	AMH Gen II ELISA	Access AMH assay	Elecsys AMH Immunoassay	Ultra-Sensitive AMH/MIS ELISA	MenoCheck picoAMH ELISA
**Lower limit of detection (LoD)**	0.08 ng/ml	0.02 ng/ml	0.01 ng/ml	0.023 ng/ml	1.3 pg/ml
**Lower limit of quantitation (LoQ) with <20% CV**	0.17 ng/ml	0.08 ng/ml	0.03 ng/ml	0.06 ng/ml	3.2 pg/ml
**Intra-assay coefficient of variation**	<=5.4%	<=1.7%	<=2.6%	<=4.0%	<=5.5%
**Inter-assay coefficient of variation**	<=5.6%	<=2.8%	<=3.9%	<=4.8%	<=8.1%
**Calibration points**	7 points	6 points	2 points	6 points	6 points
**Sample volume**	20 µl	20 µl	50 µl	25 µl	100 µl
**Assay time**	>3 hours	40 minutes	18 minutes	2.5 hours	4.5 hours

## Comparison Between the Various AMH Assay Methods

In the earlier years, a comparison between the Immunotech and DSL ELISAs showed widely different regression relationships, with lower, comparable or higher serum AMH values by Immnotech compared with DSL ELISA being reported in different studies (47–50).

Studies comparing the AMH Gen II ELISA, DSL and Immunotech AMH ELISAs (30, 50, 51) showed good correlations of AMH Gen II ELISA with both the DSL and Immunotech kits although higher numerical values were shown by AMH Gen II ELISA compared to the latter. A higher numerical value was generated by the AnshLabs assay compared with the Gen II assay (43, 50–52).

AMH values obtained with the Gen II kit were well correlated with those generated by the Access and Elecsys^®^ automated immunoassay methods, with correlation coefficients being >0.9 (p<0.001) in all pairwise comparisons (52). Passing and Bablok regression revealed that the values generated by the Access AMH assay were comparable to those generated by the Gen II assay, whereas those generated by the Elecsys AMH Immunoassay were systematically lower (**Supplementary Figure 2**). The bias between the Beckman-Coulter platforms (Gen II assay and Access AMH Assay) and the Elecsys AMH Immunoassay was uniform across the whole range of values studied. The finding concurred with previous reports, although the Elecsys AMH Immunoassay was claimed to be standardized against the Gen II assay (53–57). This differential calibration should be kept in mind when results generated by the different assay methods are interpreted or compared in clinical practice or research settings.

## Assay Stability Upon Different Sample Storage Conditions

In the Access AMH assay and Elecsys AMH Immunoassay, serum samples frozen at -20°C and -80°C gave significantly lower AMH values (p<0.05) compared with freshly collected samples (52), with significantly lower values for those stored at -20°C compared with -80°C (p<0.05). The magnitude of the difference between immunoassays is small (<0.2 ng/ml) and may not be clinically important. The basis for this loss of immunoactivity with frozen storage remains to be explored.

## Development of an International AMH Reference Reagent for Immunoassay

There has long been an unmet need for an internationally available reference material for AMH. This would allow calibration of diagnostic AMH immunoassays against a standard, which would then allow values obtained from different assay systems to be compared. An international collaborative study (7, 8) was thus undertaken by the World Health Organization (WHO) to produce a reference material purified from media from a stable human ovarian cancer cell line and available in ampoules (coded 16/190), and to derive an immunopotency evaluation for this human AMH preparation. The AMH preparation consisted of the full length 140kDa form with a modification of the internal cleavage site to ensure maximum cleavage between the pro-hormone and mature forms of the molecule (58) which represent the major forms of AMH in human serum (14, 15, 32). The WHO study involved the distribution the WHO reference preparation to participant laboratories, along with 21 human serum samples of varying origin. Each participant laboratory was requested to include these samples in their own immunoassay system using the AMH reference preparation of their own kit. Study participants used 21 different assay methods, 19 of which were different methods/platforms (either manual or automated) combinations. Since there is currently no recognized AMH preparation to act as an international reference, the results for serum values and the WHO reference reagent varied markedly between assays. The immunopotencies of the WHO reference reagent for the 21 laboratories ranged from ~350 to ~1200 ng/vial. In order to develop a consensus potency for the WHO Reference reagent, results of those 16 methods which were statistically comparable were combined to yield a robust geometric mean of 489 ng/ampoule. In addition, the bias of individual serum AMH samples for all kit results from the consensus means were also determined. In parallel, the bias attributed to the WHO AMH reference reagent in individual assays from the consensus mean was determined and compared with the corresponding bias observed with the serum samples.

Interestingly, in many of the assays the bias for the WHO AMH reference reagent within assay was statistically dissimilar to that observed with serum samples. Thus, while the use of the WHO AMH reference reagent as an International reference preparation should reduce the variability between some assays, it is apparent that the WHO AMH reference reagent is not being recognized in a similar manner to serum samples in all methods. The reason for the dissimilar responses between serum and WHO AMH reference reagent in these assays is not apparent (8). Clearly, the selection of kit reference preparation used in these methods is important, but other explanations regarding the different methodologies can be considered. From a global perspective one should not be too surprised by these results. AMH is a large complex glycoprotein which is found in the circulation in both precursor and processed forms (14, 15, 32). Little is known of the various heterogeneous AMH forms found in serum and it is unclear to what extent these forms are comparable with the purified recombinant preparation used as the reference reagent. Recently, AMH isoforms have been identified in human follicular fluid and granulosa cell extracts which do not match recognized consensus forms, suggesting that additional, as yet unknown, processing occurs (59). Additionally, the choice of antibodies used in the respective assays is also critical. Immunoassays of this sort are comparative assays where the adage ‘like *vs*. like’ strictly applies. For a serum assay, the most appropriate reference preparation should be serum-based, reflecting the samples under investigation, and yet the matrix used in the 16/190 preparation was bovine casein-based instead of human serum-based. The question of using a serum pool as a reference preparation was discussed by Ferguson and colleagues (8) but was not progressed due to problems of availability, standardization and continuity of a pooled serum standard supply. In contrast, the WHO AMH reference reagent satisfies many of the requirements expected of an international reference preparation and is the first widely available, stable, lyophilized preparation of AMH that can be used for harmonization of the current clinically relevant immunoassays. Its introduction should lead to greater consistency between the different kit assays. However, although the WHO reference reagent is likely to be commutable in a proportion of AMH assays, commutability with clinical samples has been demonstrated only in some but not all assays. As such, the reference reagent may not effectively harmonize the results for clinical samples in all assays, and because of this, has not been established as a WHO International Standard. Instead, its status as a WHO reference reagent represents its intended use as a common material with which manufacturers can investigate assay performance characteristics. This is critical as a first step in the continuum toward eventual AMH assay harmonization and will likely pave the way for a second generation of reference material(s) with which a more universal demonstration of commutability with clinical samples will be possible.

## Discussion

AMH immunoassays are now widely available for assessing ovarian reserve and have application in a number of reproductive conditions where the size of the ovarian reserve is clinically important. Immunoassays are now available both in automated and manual formats with the automated platforms showing superior assay characteristics. In addition, new sensitive immunoassays are now available for situations where AMH serum concentrations are low, as seen in young women following chemotherapy and in women approaching menopause.

At recent count there are AMH kits provided by more than 14 manufacturers, most with their own AMH reference preparations which to date have not been calibrated against a common (international) reference preparation. The WHO AMH Study (8) was an attempt to establish such an appropriate reference preparation to aid in the harmonization of assay results. One of the primary goals in the harmonization of immunoassays is that, for a given analyte, the same numeric result should be obtained for a clinical sample irrespective of the assay method used to derive that result. This facilitates the derivation and effective use of clinical practice guidelines and supports evidence based medicine. A lack of harmonization can lead to different methods providing divergent results for the same clinical sample and clinicians and other healthcare professionals, who are often unaware of these differences, may wrongly classify a patient’s health status. Central to improving agreement between the results of different assay methods is the traceability of calibration to reference preparations and there is now acceptance that these reference preparations should be commutable, i.e. they should behave in the same way as the native analyte itself. The mathematical definition of commutability in effect states that for two samples (e.g. test and reference), the ratio of the results from the samples will be the same for each assay method. Based on the lack of commutability of the WHO AMH reference reagent with serum samples in some assay methods, this preparation cannot be considered as a suitable universal immunoassay reference preparation, although it will play an intermediate role while a second generation preparation is identified. Identification of that preparation will require knowledge of serum forms of AMH so that a suitable compatible reference preparation can be identified. The current challenges of creating an international reference preparation are summarized in **Table 2**.

**Table 2:** Challenges in creating a valid International reference preparation.

Considerations in the preparation of an International Standard	Challenge	Desired Outcome
Identifying a common reference preparation suitable in multiple diagnostic assays with differing specificities	Many circulating forms of AMH exist in the circulation. Although assays report that they detect and quantify the same target, they often have different specificities for those different circulating forms and so the preparation of a standard that is suitable for all assays becomes very challenging	The preparation of a standard that contains a “representative” mixture of all circulating forms may not be sufficient. It may be more appropriate to prepare separate standards for each circulating form. However, this may not have wide acceptance with all end users.
		
Choice of reference material e.g. plasma/serum-based, synthetic or recombinant i) effect on commutability; ii) sample volume and concentration	Concept of “like versus like” (reference material should behave in the same way as the samples being analysed) is especially relevant to the commutability of a reference material with clinical samples An International Standard is usually prepared as a large batch of vials or ampoules to be available for >10 years to prevent the need for end-users to regularly recalibrate their assays. The preparation of such a reference standard may require the pooling of patient samples to obtain sufficient material.	Since the general principle is that of “like versus like”, often a standard is made using plasma or serum. Pooled plasma or serum with recombinant protein may be appropriate, but the requirement remains for the behavior of the standard to be the same as that of the test samples and that the standard is commutable with clinical samples across all assays. The pooling of patient samples or purification of the analyte from its native matrix or the substitution of the native analyte with a non-native version (e.g. recombinant) are manipulations that can change the nature of the standard to render it no longer commutable with patient samples. This is evaluated in a multi-method international collaborative study
		
Long term stability of reference material – effect on commutability	International Standards are expected to be stable for >10 years to prevent the need for regular recalibration exercises which can be expensive and problematic for end users. In addition, these materials must be shipped globally.	For these scientific and logistical reasons, the material needs to be formulated with specific stabilizing excipients and is usually lyophilized and potentially further altered in comparison to the native specimen matrix. The effects of these manipulations on the commutability of International Standards must be evaluated in the collaborative study. It may be possible to prepare a standard using unadulterated frozen material but the challenges associated with the long term stability and stability-on-shipping often prevent this approach.

The reader is referred to the WHO document ‘Recommendations for the preparation, characterization and establishment of international and other biological reference standards’ (60) for a fuller description.

## Author Contributions

HL and WL conceived the idea of this article. HL and DR conducted literature search and wrote the manuscript, with critical input from CB and WL. All authors contributed to the article and approved the submitted version.

## Conflict of Interest

The authors declare that the research was conducted in the absence of any commercial or financial relationships that could be construed as a potential conflict of interest

## Supplementary Material

Supplementary Figure 1 Multiple forms of AMH generated by post-translation cleavage. The amino acid sequence is numbered from the N-terminus of the pre-proAMH in this figure. The proAMH molecule itself contains 230 amino acid residues.

Supplementary Figure 2 Correlation between the Beckman-Coulter Gen II assay (Gen II), Access AMH assay (Access), and Elecsys AMH Immunoassay (Roche) for determination of AMH (n=94). The upper panels represent the Passing and Bablok regression plots whereas the lower panels represent the Bland-Altman plots. (Reproduced with permission from Li et al., 2016) (50).
