# Supplementary material for: Challenges in Measuring AMH in the Clinical Setting
Source: Front Endocrinol (Lausanne). 2021 May 24;12:691432. doi: 10.3389/fendo.2021.691432 (PMC8183164; doi:10.3389/fendo.2021.691432)
Supplement: Supplementary Figure 1 — Multiple forms of AMH generated by post-translation cleavage. The amino acid sequence is numbered from the N-terminus of the pre-proAMH in this figure. The proAMH molecule itself contains 230 amino acid residues. [file DataSheet_1.pdf]

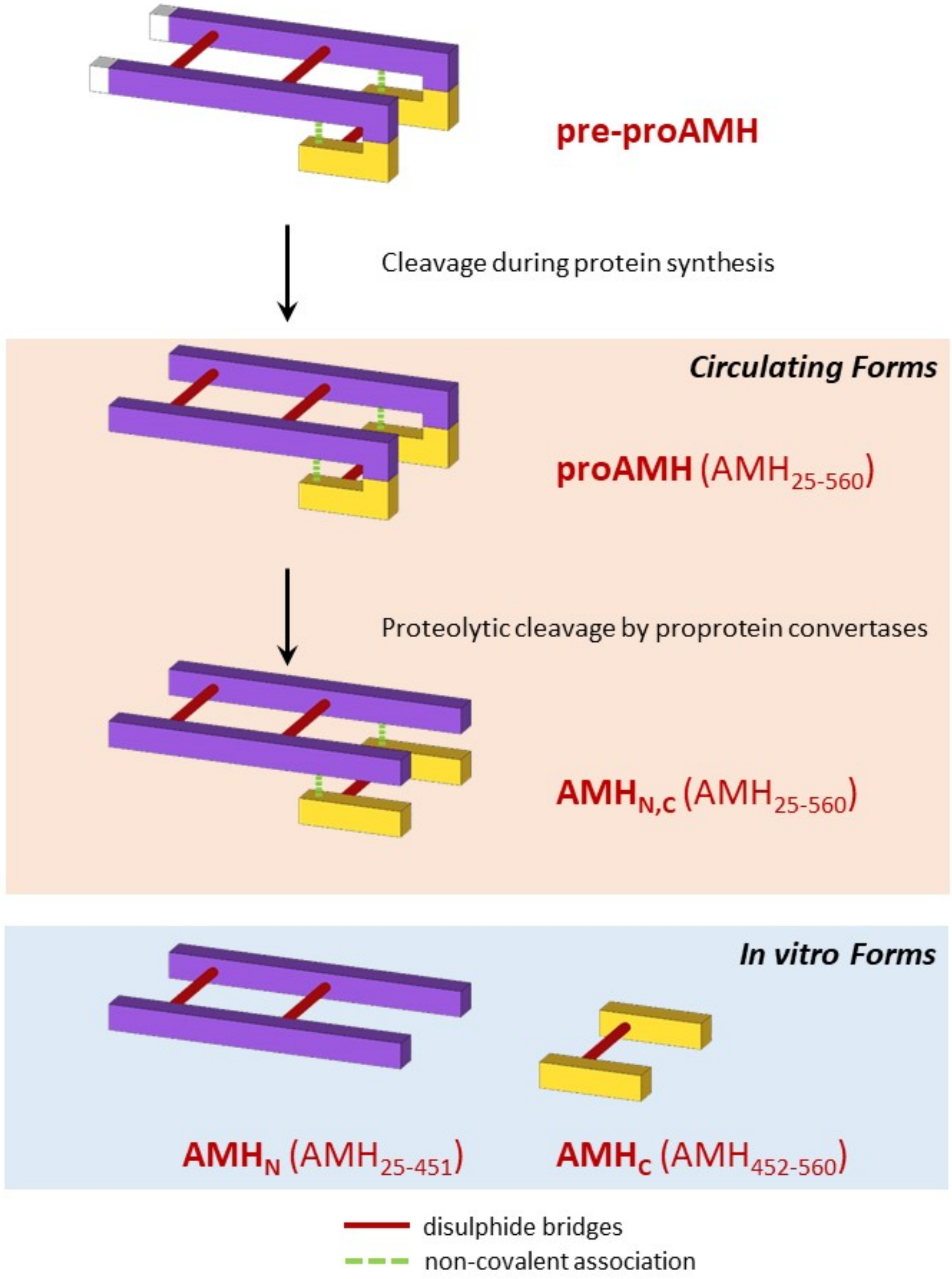
pre-proAMH
Cleavage during protein synthesis
Circulating Forms
proAMH (AMH25-560)
Proteolytic cleavage by proprotein convertases
AMHN,C (AMH25-560)
In vitro Forms
AMHN (AMH25-451)
AMHC (AMH452-560)
disulphide bridges
non-covalent association
