# Supplementary figures and images for: Challenges in Measuring AMH in the Clinical Setting
Source: Front Endocrinol (Lausanne). 2021 May 24;12:691432. doi: 10.3389/fendo.2021.691432 (PMC8183164; doi:10.3389/fendo.2021.691432)

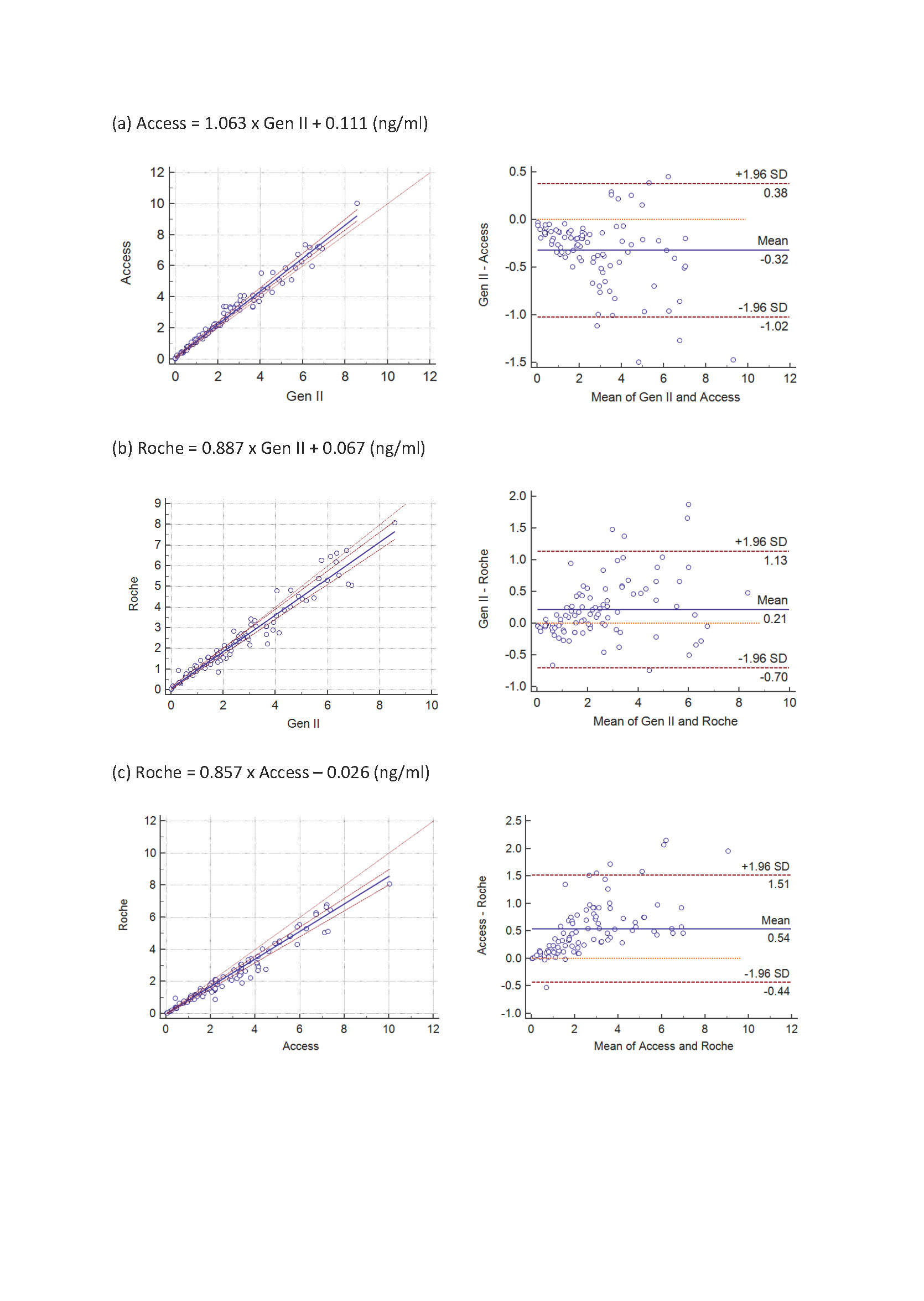

Supplement: Supplementary Figure 2 — Correlation between the Beckman-Coulter Gen II assay (Gen II), Access AMH assay (Access), and Elecsys AMH Immunoassay (Roche) for determination of AMH (n=94). The upper panels represent the Passing and Bablok regression plots whereas the lower panels represent the Bland-Altman plots. (Reproduced with permission from Li et al., 2016) (50). [file Image_1.jpeg]
